# Evaluating the effectiveness of conservation and development investments in reducing deforestation and fires in Ankeniheny-Zahemena Corridor, Madagascar

**DOI:** 10.1371/journal.pone.0190119

**Published:** 2017-12-21

**Authors:** Karyn Tabor, Kelly W. Jones, Jennifer Hewson, Andriambolantsoa Rasolohery, Andoniaina Rambeloson, Tokihenintsoa Andrianjohaninarivo, Celia A. Harvey

**Affiliations:** 1 Betty & Gordon Moore Center for Science, Conservation International, Arlington, Virginia, United States of America; 2 Human Dimensions of Natural Resources, Colorado State University, Fort Collins, Colorado, United States of America; 3 Conservation International Madagascar, Antananarivo, Madagascar; Universita degli Studi della Tuscia, ITALY

## Abstract

Forest conservation and REDD+ projects invest millions of dollars each year to reduce local communities’ dependence on forests and prevent forest loss and degradation. However, to date, there is limited evidence on whether these investments are effective at delivering conservation outcomes. We explored the relationships between 600+ small-scale conservation and development investments that occurred from 2007 to 2014 and conservation outcomes (deforestation rates and fire detections) within Ankeniheny-Zahamena Corridor in Madagascar using linear fixed effects panel regressions. We derived annual changes in forest cover and fires from satellite remote sensing. We found a statistically significant correlation between presence of any investment and reduced deforestation rates in 2010 and 2011 –years with accelerated deforestation elsewhere in the study area. This result indicated investments abated deforestation rates during times of political instability and lack of governance following a 2009 coup in Madagascar. We also found a statistically significant relationship between presence of any investment and reduced fire detections in the study area, suggesting investments had an impact on reducing burning of forest for agriculture. For both outcomes (i.e., deforestation rates and fire detections), we found that more dollars invested led to greater conservation outcomes (i.e. fewer fires or less deforestation), particularly when funding was sustained for one to two years. Our findings suggest that conservation and development investments can reduce deforestation and fire incidence, but also highlight the many challenges and complexities in assessing relationships between investments and conservation outcomes in a dynamic landscape and a volatile political context.

## Introduction

Deforestation and forest degradation continue to threaten tropical forests, their biodiversity, and the ecosystem services they provide. International donors and development agencies have spent billions of dollars in Africa to curb deforestation since the 1990s, with little evidence on the impact of these investments [1]. More recently, REDD+ (Reducing Emissions from Deforestation and Forest Degradation plus the enhancement of forest carbon stocks, sustainable management of forests, and conservation of forest carbon stocks) has increased investments in Africa and globally to mitigate climate change [2]. Based on United Nations Framework Convention on Climate Change guidance to implement REDD+, countries need a national REDD+ strategy, a national forest monitoring system, a forest reference emissions level or forest reference level, and a system for implementing and providing information on safeguards [3,4]. If a country is not yet able to demonstrate emissions reductions at the national level, subnational forest reference levels and monitoring and reporting systems can be recognized on an interim basis. Countries can choose to include REDD+, and associated emissions reductions, in their Nationally Determined Contributions and implement associated forestry sector activities at the national, regional, and community levels. These activities may include establishing protected areas under a range of management scenarios from strict enforcement to community forest management, promoting sustainable forest management, and restricting the use of fires and/or promoting alternative livelihoods for communities to reduce pressure on remaining forests, among others. A critical component of REDD+ financing is that projects demonstrate ‘additionality’ in the benefits the project provides to reducing deforestation or forest degradation; that is, projects are expected to deliver additional reductions in greenhouse gas emissions beyond what would have happened in the absence of the project activities [5].

There is a growing concern in conservation financing that many of the projects and investments intended to reduce deforestation and forest degradation have not been rigorously evaluated [6–7]. In addition, there are concerns that investments may result in perverse outcomes such as leakage of deforestation to other areas [1,8]. While there have been recent calls for more rigorous evaluation methods that account for the causal relationships between conservation investments, including REDD+, and deforestation outcomes (e.g., [9–12]), these assessments are not always easily applied to conservation projects due to their multiple outcomes and scales, and the large number of confounding factors that affect outcomes [13]. Additional challenges to rigorously evaluating the effects of conservation investments include a lack of baseline data on forest cover and deforestation rates, and data on interventions or outcome variables at the appropriate spatial or temporal scales [7,9]. To date, most impact evaluation studies have focused on evaluating the effectiveness of protected areas [14–17] or payments for ecosystem services programs [18–22] on forest cover outcomes. Much less is known about the effectiveness of forest conservation initiatives such as REDD+ [but see 12,23–25].

During colonial rule, Madagascar lost 70% of its forest cover by resource exploitation in just thirty years between 1895 and 1925. Today, the fragmented and narrow corridors of primary forest that remain are critical to rural livelihoods, but are under threat from subsistence farming practices that are legacies of colonialism [29]. Madagascar has received significant investments in REDD+ [1,8,26–28] due to its carbon-rich forests and high rates of deforestation. Ankeniheny-Zahamena Corridor (known as CAZ, for its French acronym) is one of the largest remaining humid tropical forest patches in Madagascar [30] and a focus for REDD+ investments since 2007. In addition to representing a biodiversity and species endemism hotspot, CAZ provides critical ecosystem services of both local and global importance. CAZ provides freshwater to over 350,000 inhabitants living within its watersheds as well as hydroelectric power to the nation’s capital, Antananarivo, and the provincial capital, Toamasina [31–32]. The carbon-rich forests also provide a valuable global ecosystem service, storing an average total carbon stock of 166.3 Mg C ha^-1^ [33]. While the traditional practice of using fire to clear land for swidden agriculture supports rural livelihoods, this practice continues to threaten the corridor’s remaining forests even though CAZ was proposed as a protected area in 2005 and officially designated in 2015 [34–36].

In 2007, a REDD+ pilot project was initiated in CAZ to reduce 8 million tons of CO_2_ emissions by 2017 [32]. The 370,000 ha project site included two zones—a rigorously managed, strict conservation zone with no inhabitants, and a buffer zone with settlements in which sustainable forest use (e.g., fuel wood extraction, timber, non-timber forest products) is permitted [32]. As part of the REDD+ project, hundreds of small-scale conservation investments were implemented to reduce deforestation and mitigate fire use in CAZ, beginning in December 2007 [32]. These investments included conservation activities such as community forestry management, forest patrolling, and conservation agreements, as well as small-scale livelihood projects (such as beekeeping, fish farming, agricultural production, etc.) that aimed to improve local livelihoods while reducing pressure on local forests from communities. After an independent verification, the REDD+ project was formally validated in 2013 [37]. While conservation and development projects have been a critical part of the REDD+ pilot project and other forest conservation initiatives in the region, until now, no one has assessed whether these investments had the intended outcomes of reducing deforestation and forest fires in the region.

The objective of our study was to analyze whether REDD+ investments in conservation and development projects within CAZ have led to conservation outcomes. Specifically, we examined whether the number of projects or amount of financial investment was related to a reduction in deforestation and/or a reduction in fires. Our analysis of investments used a novel and comprehensive database of investments in CAZ from 2007 to the end of 2014. We combined these data with remotely sensed datasets on forest cover change and fire detections during the same time period. This analysis contributes to the scant evidence base on whether REDD+ projects have led to conservation outcomes and highlights the complexities of relating REDD+ investments to conservation outcomes in a dynamic and volatile landscape. We make recommendations on how future evaluations of REDD+ and forest conservation programs can improve data collection and evaluation design efforts to provide robust scientific evidence on the conservation impacts of investments.

## Materials and methods

### Study area

**Fig 1:**
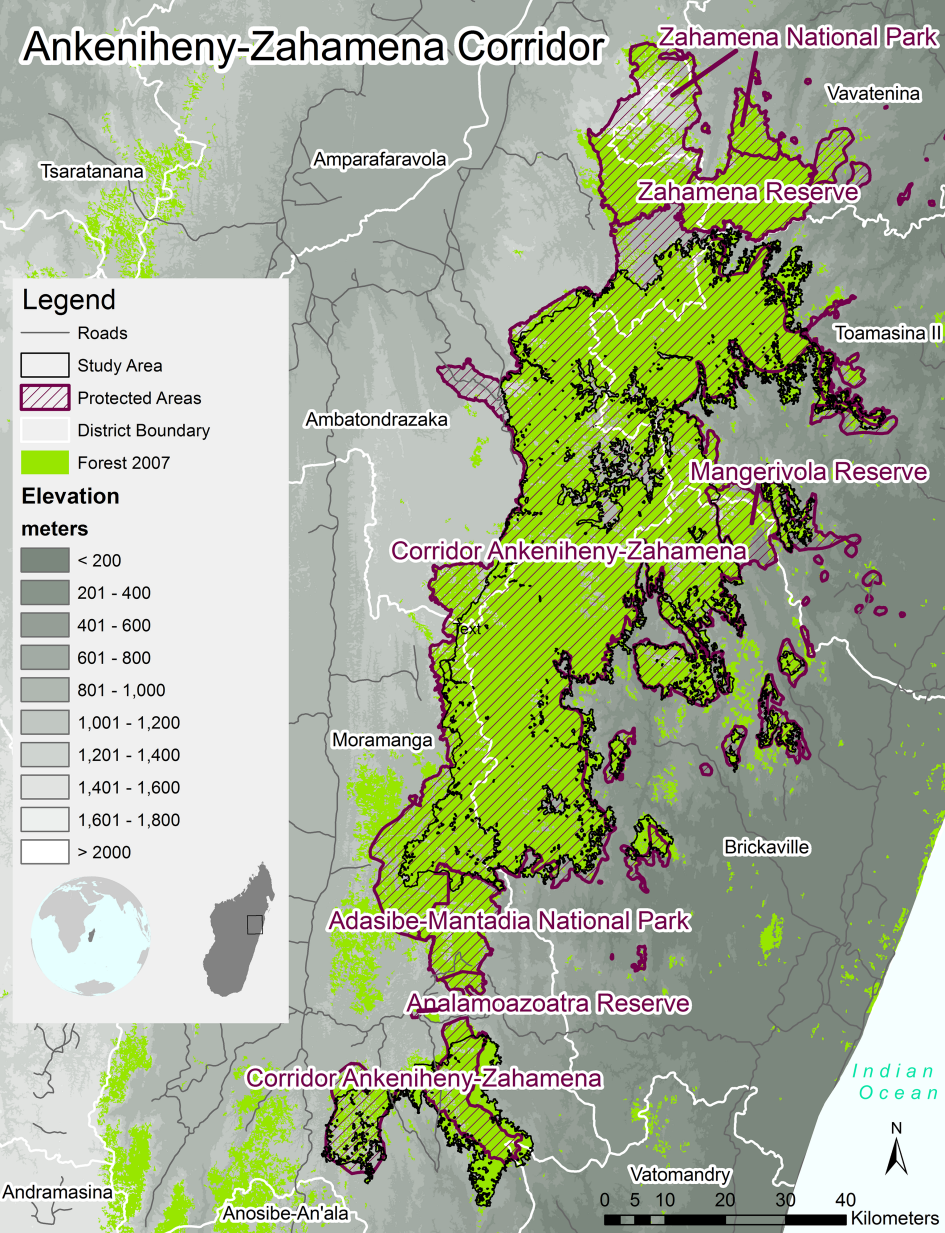
The CAZ study area. The study area, outlined in black, was based on the REDD+ project area and was defined as only primary forest in circa 2007.

CAZ is located in central eastern Madagascar and contained 370,000 ha of intact, moist humid forest encompassed by a mosaic of fragmented forests, crops, fallows, and rice paddies in 2007, the year REDD+ activities began [32]. We defined our study area using the boundaries of the CAZ REDD+ project area. The CAZ REDD+ project area only included mature forest (>7 m in height and > 80% canopy closure) in circa 2007 ([30,32]; Fig 1). The study area was contained within the designated CAZ protected area boundary. A 1,000 m elevation gradient exists within the CAZ landscape, ranging from 200 m above sea level on the eastern boundary to 1,300 m on the western boundary. The lower elevation in eastern CAZ increases forest accessibility; this lower elevation also facilitates rainfed rice and maize production, both associated with swidden agriculture practices.

Madagascar is administratively divided into four main political units: regions, districts, communes, and fokontany. Our study area overlapped with 88 fokontany, 25 communes, 5 districts, and 2 regions [38]. According to the 2008–2009 Madagascar census, 114,000 people lived in the 88 fokontany that intersected the study area, although we cannot determine how many lived inside the study area boundary due to the lack of detailed maps of villages and communities [39]. The majority of the population in the CAZ area are subsistence farmers who cultivate rice and other staple crops. Most farmers live below the national poverty levels and are food insecure [40].

We examined the impact of investments from the beginning of 2007 (the year REDD+ activities began) through the end of 2014, using all available information on these investments. This timeframe included a period of political instability surrounding the 2009 political coup and two political elections in December 2006 and December 2013. These events have been associated with acceleration in deforestation nation-wide resulting from the lack of government oversight and limited resources available for forest protection [41–42].

### Investments database

We meticulously collected details on all investments made in CAZ from 2007 to 2014, using reports, financial records, and interviews with staff from Conservation International Madagascar, national Non-Governmental Organizations, and international agencies. From 2007 to 2014, CAZ received >600 investments from a variety of different organizations and projects, all in support of forest conservation and rural development. The majority of the mapped investments in our study (83%) were aimed at improving local livelihoods and reducing their dependency on destructive forest practices, particularly the clearing and burning of forest to establish new agricultural areas [43]. The other 17% of investments included direct conservation activities such as community forestry management, forest patrolling, and conservation agreements; all specifically designed to reduce deforestation and fire incidence.

Projects were delivered under a variety of different approaches, including incentives, direct implementation, grants, social safeguards, and general conservation activities referred to as “other” (Table 1). The landscape of mixed investments in CAZ was similar to other REDD+ investment strategies that build off existing conservation and rural development efforts while also applying alternative approaches to incentives [44]. Inconsistencies in documentation in both the types of investments as well as the implementing agencies proved a major challenge to data collection, particularly spatial data on the location of the specific communities that received investments. For this reason, the investments could only be accurately mapped to the fokontany that contained the communities that received investments. While we documented a total of 629 investments, only 553 of those investments could be mapped to an individual fokontany; we mapped the remaining 76 investments to the commune-level. We used expert local knowledge from Conservation International Madagascar staff working in CAZ to fill in gaps in the spatial and temporal information of partner organization investments data as well as missing spatial information in the financial and project reports.

**Table 1:** Characteristics of the conservation and development investments made in Ankeniheny Zahamena Corridor, Madagascar, from 2007 to 2014.

Type of investment	Description	Number of invest- ments in study area	Total invest-ments (USD)^1^	Median invest- ment per hectare of forest c.2007 (USD)	Mean invest-ment (USD)	Standard deviation invest- ment (USD)	Percent of projects with financial infor- mation	Average project duration (months)	Standard deviation project duration (months)
Incentives	Payments or non-monetary rewards for the successful implementation of livelihood projects	144	256,305	0.62	1,913	1,586	93%	15	14
Direct Implement-ation	Conservation activities funded through the International Development Assistance credits from the World Bank and implemented by Conservation International Madagascar	74	77,391	0.76	1,060	723	100%	9	2
Grants	Monetary grants awarded to organized communities (known locally as VOI) [45] with management authority over forests, interested in a variety of conservation activities (i.e. forest restoration, forest management, and training in beekeeping and farming)	237	651,478	0.77	2,820	2,290	97%	13	8
Social Safeguard	Payments for sustainable livelihoods to communities potentially experiencing a negative livelihood impact from the establishment of the CAZ protected area were only implemented between January and June of 2014	53	955,553	0.95	18,376	26,368	100%	7	1
Other	A variety of conservation activities (i.e. active patrolling, capacity building for natural resource management) implemented by national Non-Governmental Organizations with funding from the World Bank and USAID	123	N/A	N/A	N/A	N/A	0%	50	37
**All**		**629**	**1,940,727**	**0.77**	**3,961**	**10,033**	**78%**	**20**	**24**

^1^ Investments are in US dollars (2014)

Our final investment dataset included the number of investments each year, length of investments, and total investment amount (US dollars) (Table 1) [46]. We were unable to collect financial information on the “other” investments and a few activities related to incentives and grants (22% of the database) due to the lack of available financial records. We did not include CAZ-wide investments that occurred during our study period because we assumed these projects were invested equally across CAZ. These included various USAID investments, through the National Environmental Plan, that started in 1984 and continue today through a variety of market expansion, community-based forest management, food security, health, and protected area governance mechanisms [47–49].

### Deforestation dataset

#### Mapping deforestation

We estimated annual deforestation rates by visually interpreting Landsat data from 2005 to 2014 and digitizing forest change directly between each year of the study [50–51]. We chose this direct change mapping method to utilize a free and accessible data source (Landsat) and thus enable cost-effective evaluations. To increase the detection of small-scale deforestation and forest fragmentation, we pan-sharpened the multi-spectral Landsat (30 m) to 15 m using the 15 m panchromatic band and a Brovey transform with bilinear interpolation in Erdas Imagine version 2014 [52]. We used a total of 23 scenes of Landsat 7 and Landsat 8 images for path 158 row 73 and path 158 row 72 acquired from the U.S. Geological Survey (https://usgs.gov). The Landsat 7 Imagery was preprocessed with ENVI 4.7 to create gap-free composites to adjust for the scan line corrector malfunction. Using ArcGIS 10.2, we visually interpreted forest and forest loss. We used multiple images from the same year to capture areas obscured by cloud cover in one image. If no cloud-free views where available in the same year, we used the most recent cloud-free view available from previous years. We interpreted forest loss based on changes in color and texture from the previous cloud-free view of the same area and delineated the deforested areas (i.e. areas that transition from forest to any non-forest type (i.e. agriculture, water, urban, road), by digitizing polygons over the image. The minimum mapping unit for forest change was 0.1 ha (approximately 4 pixels). Similar to Harper et al. 2007 [30], we did not consider forest regrowth or secondary forest as primary forest in our analysis. Regrowth dynamics in this region can be more complicated than elsewhere in the tropics. In other parts of the tropics, rapid regrowth of secondary forest during the fallow period means there is not permanent loss of forest cover. This is not the case in CAZ where the repeat frequencies of swidden agriculture practices often results in the permanent loss of forest cover, as after multiple fallow periods, there is aggressive growth of grasses (Imperata cylindrica) and ferns that prevent forest regrowth [34, 36].

#### Accuracy assessment

We performed an accuracy assessment of our forest loss class using 15 m Aster imagery from U.S. Geological Survey. We selected Aster imagery for c.2006 (based on available 2005 and 2006 images; and c.2016 (based on 2015 and 2016 images). We used a stratified random sampling design, based on [53], to distribute validation points within both the stable forest class and the forest loss class. We calculated producer’s, user’s, and overall map accuracies [54] for “stable forest 2006–2016” and “forest loss 2006–2016”. Producer’s accuracy represents the chance that any place on the ground is accurately represented by the map, while user’s accuracy captures the reliability of the map in terms of the map class correctly reflecting the real condition on the ground.

### Fire data

Globally, fire is a key driver of tropical deforestation and forest degradation [55] and remotely sensed fire data can be used as a proxy for deforestation for evaluating conservation outcomes [17,56]. Fire is the dominant driver of deforestation in Madagascar’s eastern humid forests where most deforestation and forest degradation in the region is from the clearing and burning of forests to establish agricultural systems to support people’s livelihoods [36, 57–58]. Therefore, we used satellite-derived fire detections as a second measure of the impacts of investments on conservation outcome. The data are based on thermal anomalies from the MODerate resolution Imaging Spectroradiometer (MODIS) instruments [59] at a spatial resolution of 1 km. While the spatial resolution is coarse, the temporal resolution of the data (every 6 hours) is ideal for finding cloud-free observations in this region. To examine fire incidence across the study region, we clipped the daily 1 km fire detections from 2007–2014 to the 2007 forest cover extent (our study area boundary), and summed annual fire detections for each fokontany. For data quality control, we excluded fire detections with <30% confidence, defined by Giglio [59] as low confidence.

### Estimation method

The variability in investments across fokontany and year allowed us to use differences across space and time to detect a relationship between investments and conservation outcomes. We used linear fixed effects panel regression to estimate the relationship between investments and conservation outcomes from 2007 to 2014 [60–62]. Our dependent variables are defined as (a) annual rate of deforestation or percent change in forest cover and (b) the number of fires detected. When percent change in forest cover was the dependent variable, our panel regression equation for fokontany, *i*, in year *t*, was:

$${Defor}_{it}=\alpha \;+\;\beta_{1}C_{it}+\beta_{2}T_{t}+\;\sigma_{i}+\;\varepsilon_{it},$$ (1)

where *C*_*it*_ denotes the conservation and development investments, *T*_*t*_ is a vector of year effects, and *σ*_*i*_ + *ε*_*it*_ is the composite unobservable. This unobservable includes an individual time-invariant fixed component (*σ*_*i*_) and a time-varying component (*ε*_*it*_). *σ*_*i*_ controls for any fokontany characteristics that were time-invariant during our study period. This includes slope, elevation, distance to roads, distance to towns, and area under strict protection, which reduces the potential for omitted variable bias that occurs in cross-sectional estimation. *T*_*t*_ controls for any changes over time, such as changes in political regime, that affected all fokontany concurrently during our study period. Given the skewed distribution of percent deforestation (S1 Table and S1 Fig), we log-transformed the dependent variable prior to including in regression analysis. We estimated Eq 1 as a linear regression model based on graphs of the relationships between investments and deforestation (S2 Fig). It is common practice to treat percent data as a continuous variable in analysis, since they can take on any number between zero and 100. We controlled for serial correlation by estimating cluster robust standard errors, clustering at the fokontany-level [63].

The number of fire detections is also treated as a continuous variable in panel regression analysis. The following linear, fixed effects panel regression was estimated when fire was the dependent variable

$${Fire}_{it}=\alpha \;+\;\beta_{1}C_{it}+\beta_{2}T_{t}+\beta_{3}X_{it}+\;\sigma_{i}+\;\varepsilon_{it},$$ (2)

where *C*_*it*_, *T*_*t*_, *σ*_*i*_ + *ε*_*it*_ are as described above. *X*_*it*_ controls for total forest area. Given the skewed distribution of number of fires (S1 Table and S3 Fig), we log-transformed it prior to including in regression analysis. We estimated Eq 2 as a linear regression model based on graphs of the relationships between investments and fires (S4 Fig). We controlled for serial correlation by estimating cluster robust standard errors, clustering at the fokontany-level [63].

In Eqs 1 and 2, we defined investments, *C*_*it*_, two ways: (1) as a binary variable where “1” referred to receiving any investment in that year and “0” referred to no investment in that year; and (2) the dollar amount invested in that year in USD. All dollar investments were converted to 2014 USD. Given the skewed distribution of dollars invested (S1 Table and S5 Fig), we log-transformed dollars prior to including them in regression analysis. We explored whether the types of investments (i.e. incentives, grants, etc. from Table 1) influenced conservation outcomes differently but did not find any statistically significant differences across investment type; thus, we report results using all investment types jointly.

While we did not explicitly test whether length of investment affected outcomes, we did take length into account through the inclusion of lags of up to three years. Specifically, we tested to see if either measure of investments, *C*_*it*_, had a lagged effect on conservation outcomes by considering the relationship between investments made one to three years before the outcome variable. We examined potential lagged effects because it is possible that some investments (such as investments in agricultural productivity) may only have an effect on conservation outcomes a year or more following the investment (e.g., if farmers experience significantly higher crop yields in a year, due to the project investment, they will potentially clear less forest land in the next year as they can produce sufficient rice on their existing land). The general panel regression equation estimated was:

$$Y_{it}=\alpha \;+\;\beta_{1}C_{it}+\beta_{2}T_{t}+\beta_{3}X_{it}+\;{\beta_{4}C_{it-n}+\sigma }_{i}+\;\varepsilon_{it},$$ (3)

where *Y*_*it*_ refers to either percent change in forest cover or fire detections as the dependent variable, and *C*_*it*−*n*_ is the lagged investments; n is the number of lags, ranging between one year and three years. We used a Wald test to test whether the contemporaneous investments, *C*_*it*_, and lagged investments, *C*_*it*−*n*_, were jointly significant in the regression. The null hypothesis in the Wald test was as follows:

$$\beta_{1}C_{it}=\beta_{4}C_{it-n}=0.$$ (4)

We used three different samples in our regression analyses. First, we ran regressions with all 88 fokontany in the study area using information on all investments (Table 1). Second, we conducted regression analysis after excluding any fokontany within a commune that we recorded as having a commune-level investment in that year but for which we could not tie the investment to a particular fokontany. We did this to ensure that we were not incorrectly assuming a relationship between investments mapped at the commune-level and fokontany-level investments. Third, we conducted regression analysis including only those fokontany that ever received an investment. Of the 88 fokontany, 61 received an investment in at least one year and 27 never received an investment between 2007–2014. We did this to address concerns about selection bias—which would occur if conservation investments were targeted at specific fokontany because of their forest cover or other characteristics [60]. For each of these three samples we also estimated regressions excluding the year 2007 since REDD+ investments did not start until December 2007.

## Results and discussion

### Deforestation accuracy

The overall accuracy for mapped deforestation over the study period was 91%. The producer’s accuracy for both stable forest and deforestation were 90% and 92% respectively. While the user’s accuracy for stable forest was 98%, the user’s accuracy for deforestation was 68%. This latter result reflects small errors of omission, likely attributable to clouds obscuring deforestation, or underestimation of deforestation.

### Trends in deforestation and fires

#### Spatial trends

**Fig 2:**
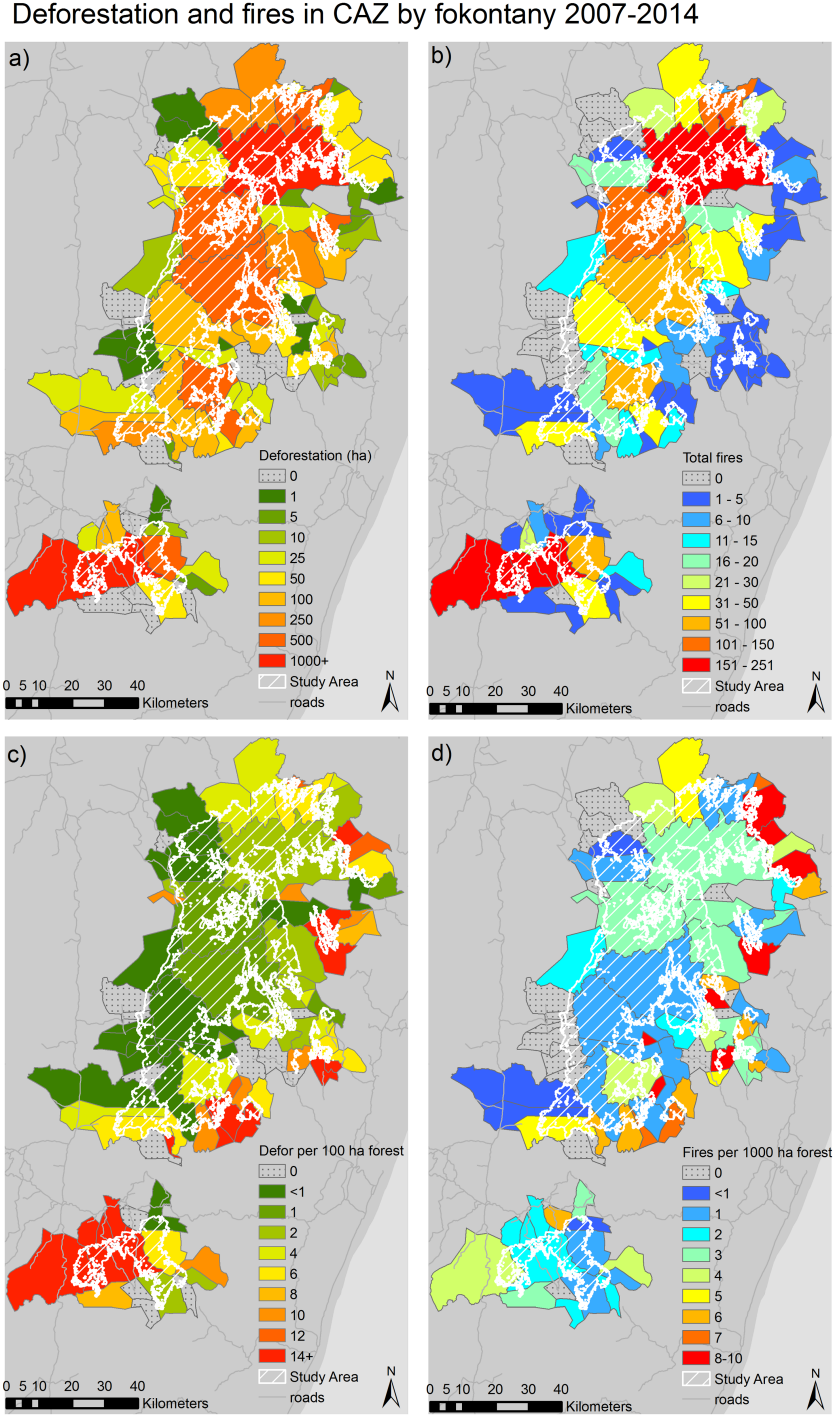
Deforestation and fires in CAZ by fokontany, 2007–2014. Spatial trends in (a) total deforestation (ha); (b) fire detections; (c) deforestation per 100 hectares of forest cover (c.2007); and (d) fire detections per 1000 hectares of forest (c.2007) in fokontany in CAZ.

Over the entire study period (2007–2014), the total deforestation and number of fire detections were highly spatially correlated (r = 0.97) indicating fires and deforestation coincided geographically in CAZ. The total hectares of deforestation and total number of fire detections in CAZ by fokontany were highest in the northern and southern extents of CAZ; and in fokontany with relatively large areas of forest remaining (Fig 2a & 2b). In 2012, the northern CAZ fokontany experienced an influx of migrants due to artisanal sapphire mining of river beds [64], and this influx of migrants may have resulted in additional clearing of forest for rice production. In contrast, the southern end of CAZ had high deforestation rates due to accessibility—this area is readily accessible from the national paved road.

High rates of deforestation and high densities of fire detections were also found in fokontany along the eastern boundary of our study area where fokontany contained less forest area than the interior of CAZ. Deforestation from swidden agriculture for rain-fed rice or maize production occurred more in this area due to accessibility and the presence of lower elevation forests [34] (Figs 1, 2c & 2d). In contrast, deforestation rates and fire detection densities were lower on the western boundary of our study area where the population is more sedentary and the main source of agricultural production is from irrigated rice fields, rather than swidden agriculture (Fig 2c & 2d). While fokontany with low rates of deforestation and low densities of fires were also situated in the middle of CAZ, it is noteworthy that deforestation and fire activity still occurred in these more remote areas. Some of this activity in the middle of CAZ may have resulted from the boom in artisanal gold mining between 2006–2009 [34] where the newly arrived miners cleared forests to plant rice and other crops to sustain their families.

Of the 88 fokontany in the study area, ten did not experience fires or deforestation during the study period. These fokontany were located along the study area perimeter and all had very little forest cover. The only exception was Ankailava, a forested fokontany, on the western boundary of the study area where communities generally practice irrigated rice production instead of swidden agriculture [34]. Further, the communities in this fokontany, are separated from the forest by a large valley which makes the forest less accessible.

A total of nine fokontany on the western border of our study area experienced deforestation but no fires were detected. However, the deforestation experienced was very small in area, less than 5 ha, and the 1 km MODIS-derived fire data were most likely too coarse to detect these small clearings. Conversely, five fokontany located along the edge of the southern patch of CAZ forest, experienced fires but no deforestation. This could also be attributed to the coarse resolution of the MODIS data, as fires detected in a 1 km^2^ area are assigned to the centroid of the 1 km pixel. It is possible, therefore, that fires occurring just outside the study area were attributed to a pixel centroid within the study area. It is also possible that the presence of fires but no deforestation in these five fokontany resulted from errors of omission in the forest loss data.

#### Temporal trends

**Fig 3:**
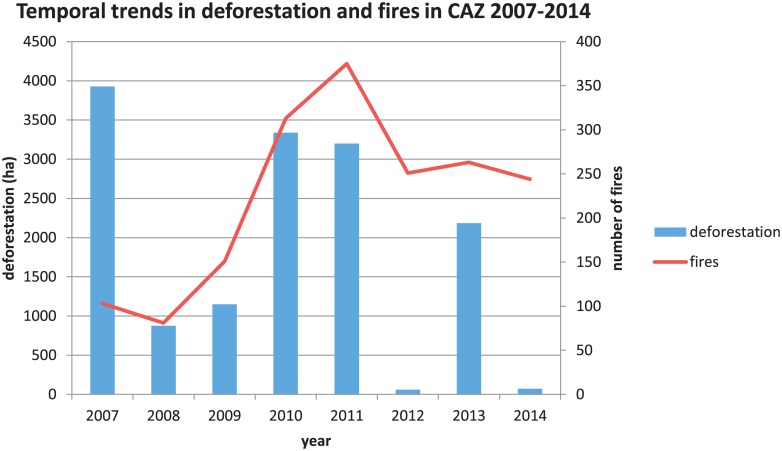
Temporal trends in deforestation and fires in CAZ 2007–2014. Annual deforestation rates (ha/year) are represented by blue bars and the number of fire detections are represented by the red line.

We assessed temporal trends in deforestation, fires, and investments for the entire CAZ from 2007–2014. Trends in deforestation and fires were similar for many (but not all) years (Fig 3). In fact, the two datasets had a low correlation (r = 0.19) for the entire annual time series from 2007–2014. When we excluded 2007 (the year when the two datasets differ the most), the correlation of the annual 2008–2014 time series increased to r = 0.61. The discrepancy in 2007 may be due to cloud persistence in the region. A year of cloud-free observations may include deforestation obscured by clouds in previous years. Deforestation and fire detections both decreased sharply in 2012 and then increased again in 2013.

**Fig 4:**
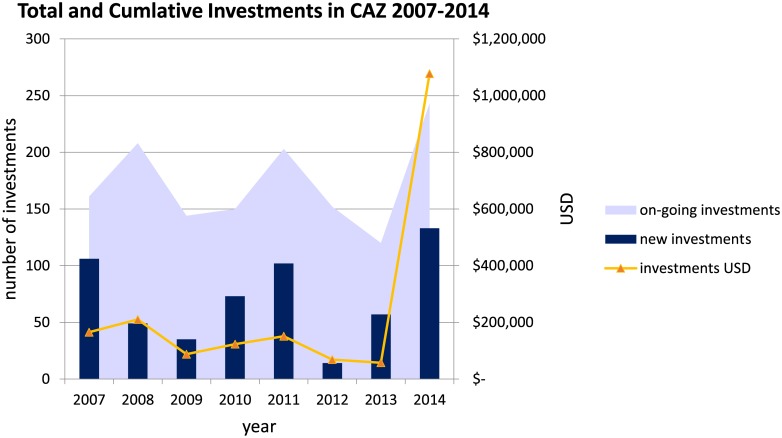
Total and cumulative Investments in CAZ 2007–2014. The number of investments between 2007–2014 in CAZ starting in each year are shown with the blue bars along with the total USD invested each year shown with orange triangle markers (USD values on the secondary y-axis). The total number of cumulative investments in any given year is shown in light blue.

The number of investments and the cumulative number of investments varied significantly across the 8 year study period (Fig 4). The number of investments is a count of new investments each year and the cumulative number of investments is a count of all on-going investments in each year. 2007, 2011, and 2014 had the highest number of new investments with 106, 102, and 133, respectively. Cumulative investments over the time period averaged 173 investments (SD of ± 38 investments) per year. Cumulative investments peaked in 2008 (208), 2011 (203), and 2014 (243). 2013 had the lowest number of cumulative investments (120).

The temporal trends in deforestation and fires highlighted annual peaks that aligned with political events. In Madagascar, fire is used to both celebrate and protest, and fire incidence is generally higher during socio-political crises or conflicts [57–58]. National elections in Madagascar occurred in December 2006 and December 2013 and in the months following these elections, deforestation increased substantially while fires showed modest increases. These trends likely reflected how in months preceding elections, officials often do not fully enforce land use policies to gain favor with local people, and administrators are often too busy campaigning to enforce regulations. Additionally, following elections, a new administration often results in staff turnover, which can also lead to lags in law enforcement [49]. 2007 was the highest year for deforestation, this essentially preceded REDD+ investment as the REDD+ activities started in December of 2007. Another large increase in both deforestation and the number of fire detections was evident beginning with the coup in 2009. From 2009 to 2012, large areas of land across Madagascar were seized and deforested without oversight or governance due to the lack of recognized government [49]. Further, many investors withdrew funding from Madagascar immediately following the 2009 coup until 2014 [65], as evidenced by the reduction in total number of investments during these years in Fig 4.

### Analysis of investment effects on deforestation and forest fires

#### Characterizing locational bias in investments

We first compared the 61 fokontany that received investments with the 27 fokontany that did not receive any investments to determine if there were any key differences in which fokontany received investments or not. The two groups of fokontany were similar in several characteristics that could be correlated with deforestation or fires (Table 2). These included: population density, percent of strict conservation zones, distance to unpaved roads or cart tracks, and distance to populated places (all p-values >0.10). The fokontany that received investments, however, had larger forest areas in 2007, higher elevations, and were located farther from paved roads, compared to those fokontany that did not receive any investments (p-values <0.05; Table 2). This suggests that remaining forested areas in CAZ were found in more remote areas and, not surprisingly, REDD+ investments tended to target areas with more forest area to protect. Similarly, of those fokontany that received investments, the fokontany with more total forest cover received, on average, more investments and larger dollar amounts of investments than those with less remaining forest. Fokontany that did not receive any investments were typically along the western side of the study area. These fokontany were located closer to major roads and contained, on average, only one third as much forest as the fokontany that received investments.

**Table 2:** Landscape characteristics and investments.

Variable (2007 values)	Fokontany that received an investment (N = 61)	Fokontany that never received an investment (N = 27)	T-value
Forest area (ha)	**5350**	**1617**	-2.51**
	*10567*	*3235*	
Percent deforestation	**1.2**	**1**	-0.31
	*2*.*8*	*1*.*7*	
Annual deforestation (ha)	**56**	**18**	-1.71*
	*164*	*40*	
Annual number of fire detections	**1.4**	**0.6**	-1.6
	*3*.*2*	*1*.*3*	
Population density	**0.25**	**0.23**	-0.65
	*0*.*24*	*0*.*11*	
Percent area under strict protection	**30**	**32**	-0.36
	*33*	*35*	
Elevation (m)	**813**	**645**	-2.54**
	*284*	*284*	
Distance to paved road (m)	**28154**	**21612**	-3.23***
	*10257*	*8026*	
Distance to unpaved road (m)	**11765**	**10310**	-1.03
	*6829*	*5743*	
Distance to cart track (m)	**3398**	**3462**	0.24
	*1671*	*1750*	
Distance to city (m)	**3949**	**3454**	-1.42
	*1419*	*1545*	
Distance to regional capital (m)	**48018**	**45865**	-0.62
	*15013*	*15058*	
Distance to national capital (m)	**152770**	**152707**	-0.01
	*23270*	*22011*	

Note: Mean in bold and standard deviation in italics. T-test values with corresponding level of statistical significance reported as:

* p<0.10;

** p<0.05;

*** p<0.01.

#### Investments and deforestation outcomes

The relationship between receiving any investment (i.e., binary variable) and deforestation rates over the 2007–2014 period was not statistically significant (Table 3). However, when we omitted 2007 from the analysis, there was a significant relationship at the 90% level between any investment and reduced deforestation for the full sample of fokontany and when only fokontany that ever received an investment were included. This correlation went away, however, when fokontany with investments mapped to the commune-level were omitted. The marginal effect of investments was around -0.1 when statistically significant and since we took the natural log of deforestation this translates to an approximate 10% decrease in percent deforestation from having an investment. Since average percent deforestation in the sample was ~1%, this is a decrease in percent deforestation of 0.1% attributable to the presence of an investment. We did not find any significant relationship between having an investment and deforestation when we used lags of one to three years.

**Table 3:** The effect of investments on percent deforestation at the fokontany-level.

	Full sample	Only investments mapped to Fokontany	Only Fokontany that received any investment	Full sample (omitting 2007 data)	Only investments mapped to Fokontany (omitting 2007 data)	Only Fokontany that ever received an investment (omitting 2007 data)
Investments measured as binary value (no lag)						
Marginal effect	-0.086	-0.006	-0.088	-0.106*	-0.063	-0.114*
Std Error	0.068	0.061	0.071	0.059	0.058	0.063
Total Observations	704	594	488	616	545	427
Total Fokontany	88	88	61	88	88	61
Investments measured as 2014 USD (no lag)						
Marginal effect	-0.003	-0.001	-0.002	-0.009	-0.007	-0.008
Std Error	0.007	0.007	0.007	0.005	0.006	0.008
Total Observations	648	545	448	567	501	392
Total Fokontany	81	81	56	81	81	56
Investments measured as 2014 USD (2-year lag)						
Marginal effect	-0.043**	-0.035**	-0.049**	N/A	N/A	N/A
Std Error	0.017	0.016	0.022	N/A	N/A	N/A
Total Observations	486	457	336	N/A	N/A	N/A
Total Fokontany	81	81	56	N/A	N/A	N/A
Prob>F-value	0.039	0.094	0.090	N/A	N/A	N/A

Note: Marginal effects from linear fixed effects regressions. Percent deforestation and 2014 USD were both log-transformed. Level of statistical significance reported as:

* p<0.10;

** p<0.05;

*** p<0.01.

N/A indicates that output with or without 2007 data is the same because data is lagged. Specifically, when estimating Eq 3, percent deforestation for the year 2007 is not included in Y_it_, because we did not have data on investments prior to 2007 (e.g., 2006 investments). Information on 2007 investments were included in Eq 3 as a lagged independent variable for 2008 outcomes (1-year lag) and 2009 outcomes (2-year lag).

When we examined the effect of presence of an investment by individual years, we found investments in 2010 and 2011 were statistically significant in reducing deforestation. In 2010, investments on average were associated with a 27% reduction in percent deforestation (p-value<0.10); this translates to a decrease in percent deforestation of 0.3%. In 2011, fokontany that received investments had, on average, a 35% lower rate of deforestation (p-value <0.05) than those that did not receive investments, or a decrease in percent deforestation of 0.4%. When we only included investments mapped to the fokontany-level, or fokontany that ever received an investment in our regressions, we found that 2011 remained statistically significant and of similar magnitude, but that 2010 was not statistically significant. These two years had some of the highest deforestation rates in CAZ; only 2007 was higher. In 2010 average deforestation was 2.4% and in 2011 it was 2.3%. The results suggest that, in these years of crisis, the fokontany that had some ongoing support and investment experienced lower deforestation rates than those fokontany where no investments were made.

When we used total dollars invested in the fokontany as the independent variable and no lags, we found no statistically significant relationship with deforestation (Table 3). When we tested the effect of lagged amounts of dollars invested we found a jointly significant effect with lags of two time periods (probability>0.04–0.1 depending on sample). This suggests that dollars invested affected deforestation when there were contemporaneous investments and previous investments over the last two years. The marginal effect was between 0.04–0.05 and statistically significant at the 95% level. Since both the dependent and independent variables were log-transformed in this regression, a 1% change in dollars invested (the independent variable) is associated with a 0.04–0.05% decrease in percent deforested. A 1% change in mean dollars spent in these fokontany is about $42.

While it is hard to know the exact reason investments did not have a consistently statistically significant effect on decreasing deforestation over all samples and tests (Table 3), there are several potential explanations. First, it could be attributable to the dynamic deforestation landscape and volatile political environment. Second, it could reflect an inability to capture and account for all investments or other types of investments that might have affected deforestation positively or negatively in the study area. Third, it could be the result of errors in the deforestation data set. Fourth, it could be missing data on other drivers of deforestation in the area that varied over space and time, such as booms in artisanal mining that led to change in population and agriculture pressure. Deforestation, especially at the fokontany-level, is likely associated with a much larger set of drivers that could be masking the effect of investments without finer-scale data on the specific location where investments took place at a village or community level. It does appear, however, that multiple years of investment are more effective (i.e., 2-year lag in Table 3) at reducing deforestation than one-off investments, and that investments may provide resilience to deforestation pressures during times of political instability.

#### Investments and fire outcomes

We found more consistent and stronger statistically significant relationships between measures of investments and the reduction of fires (Table 4). For investment as a binary measure, we found that having an investment reduced the probability of a fire by 0.14–0.16 when all years were included and about 0.18 when 2007 was excluded (p-value<0.05). After converting these marginal effects to account for the log-transformation of our dependent variable, this represented a 14–18% decrease in fire detections due to presence of an investment. There was a stronger relationship between an investment and fires when a one-year time lag was included in the regression. When two subsequent years of investments occurred, investments reduced the probability fire detections by 26–28%. When we examined the relationship between investments and fires for individual years we found statistically significant relationships in 2007, 2008, 2009, and 2012; but there was not consistency across years and samples.

**Table 4:** The effect of investments on fire detections at the fokontany-level.

	Full sample	Only investments mapped to Fokontany	Only Fokontany that ever received an investment	Full sample (omitting 2007 data)	Only investments mapped to Fokontany (omitting 2007 data)	Only Fokontany that ever received an investment (omitting 2007 data)
Investments measured as binary value (no lag)						
Marginal effect	-0.142***	-0.167**	-0.145**	-0.184***	-0.177**	-0.184***
Std Error	0.054	0.069	0.055	0.055	0.068	0.054
Total Observations	704	594	488	616	545	427
Total Fokontany	88	88	61	88	88	61
Investments measured as binary value (1-year lag)						
Marginal effect	-0.288***	-0.279***	-0.256***	N/A	N/A	N/A
Std Error	0.086	0.109	0.074	N/A	N/A	N/A
Total Observations	616	545	427	N/A	N/A	N/A
Total Fokontany	88	88	61	N/A	N/A	N/A
Prob>F-value	0.004	0.040	0.004			
Investments measured as 2014 USD (no lag)						
Marginal effect	-0.015**	-0.019	-0.013*	-0.018***	-0.019**	-0.017**
Std Error	0.006	0.007	0.006	0.006	0.007	0.007
Total Observations	648	545	448	567	501	392
Total Fokontany	81	81	56	81	81	56
Investments measured as 2014 USD (1-year lag)						
Marginal effect	-0.027***	-0.028**	-0.021**	N/A	N/A	N/A
Std Error	0.010	0.011	0.009	N/A	N/A	N/A
Total Observations	567	501	392	N/A	N/A	N/A
Total Fokontany	81	81	56	N/A	N/A	N/A
Prob>F-value	0.027	0.048	0.065	N/A	N/A	N/A

Note: Marginal effects from linear fixed effects regressions. Number of fire detections and 2014 USD were both log-transformed. Level of statistical significance reported as:

* p<0.10;

** p<0.05;

*** p<0.01.

N/A indicates that output with or without 2007 data is the same because data is lagged. When estimating Eq 3, outcomes for the year 2007 are not included in Y_it_, because we did not have data on investments prior to 2007 (e.g., 2006 investments). Information on 2007 investments were included in Eq 3 as a lagged independent variable for 2008 outcomes (1-year lag) and 2009 outcomes (2-year lag).

When we used total dollars invested in the fokontany as the independent variable and fire as the dependent variable, we found a marginal effect between 0.013 and 0.019 depending on the sample used (Table 4). When we tested the effect of lagged amounts of dollars invested we found a jointly significant effect with a lag of one time period (probability>0.03–0.07 depending on sample). The marginal effect on investments in the lagged regression was between 0.021 and 0.028. This suggests that dollars invested affected the probability of a fire occurrence. With one year of investments (no lag), the effect of a 1% change in dollars invested (about $42 USD) was associated with a 0.01–0.02% decrease in fire detections, but if there was a previous year of dollars invested (1-year lag), then investments were associated with a 0.02–0.03% decrease in fire detections.

Investments did appear to reduce fire occurrence (Table 4). Similar to deforestation, having multiple years of investment had a larger effect than just one year of investment. Many of the REDD+ investments were specifically aimed at providing alternative livelihood practices and reducing dependency on swidden agriculture, which may explain the stronger correlation between investments and reduction in fire occurrence. While deforestation and fire are generally strongly correlated, in our study area the year-to-year correlation of these two data sets was only 0.2 when 2007 was included and 0.6 when 2007 was excluded. Thus, fire occurrence data may have captured burning on existing agricultural land or young forest, in addition to burning after clearing mature forest.

#### Strengths and limitations of analysis

Our analysis is the most rigorous to date for CAZ given the large number of investments documented, the long time frame covered, and the use of two response variables (deforestation and fires) to test for conservation outcomes. While our study generated a robust database based on an exhaustive process and thorough analysis, some caveats should be considered. First, while the fixed effects method controlled for time-invariant unobservable differences in fokontany, any differences that varied over time and by fokontany were not controlled for and could have biased our regression results if the omitted variable was correlated with both investments and the outcome variable (i.e., deforestation rate or fire). For example, we did not have data on some time-varying drivers of deforestation, such as mining pressures, which accelerated in the latter years of our study period. Second, we may have missed some other types of investments that occurred in CAZ, such as rural development aid, but for which information was not available. This omission could have also biased our regressions if such investments were made and were targeted to the same fokontany as those by conservation groups, and such investments had an effect on deforestation rates or fire occurrence. Third, we did not have any data on investments prior to 2007 and these earlier investments could have influenced the impacts on conservation outcomes detected during our study period. Finally, our investment dataset was not mapped to communities or point locations, only to the fokontany-level.

Despite the limitations mentioned above, our analysis suggests that REDD+ investments can be linked to decreases in forest fires, and to a lesser extent, reductions in deforestation. It also shows that these relationships vary across years, particularly in response to larger factors driving deforestation, such as political uncertainty. Overall, we feel confident that the relationships detected indicate positive conservation outcomes from REDD+ investments in CAZ.

#### Suggestions for future conservation evaluations

While our analysis demonstrated that it is possible to identify impacts of REDD+ investments on fire detections and deforestation rates, it also highlighted the complexities of conducting these evaluations in highly dynamic forest landscapes that are typical of many locations where REDD+ and forest conservation investments are occurring. Improving the future evaluation of REDD+ and forest conservation efforts will require well-designed projects that incorporate precise location information on investments, accurate documentation of investment amounts and timing, and open and accessible records of other types of investments in the region. Additionally, attention to the spatial scale of investments and timing of data collection, plus appropriate indicator selection for measuring outcomes and confounding factors is necessary. As others have noted, for organizations to measure their outcomes and impacts requires the design of evaluation activities that are based on causal connections and clear theories of change before investment begins [9,13]. Ideally, evaluation designs will allow for control groups that do not receive investments in a landscape with similar drivers of change as groups that do receive investments, and include baseline data on conservation outcomes. However, it is also important to recognize that there are logistical, financial, and moral challenges in implementing such schemes on the ground [6,7, 66]. Conservation investments are often targeted at communities who are better organized to receive investments, as seen with grants made to Vondron’Olona Ifotony (VOI) communities, organized communities with management authority over forests in CAZ (Table 1). While targeting higher capacity communities has several intuitive advantages given the investment is made where it may have the highest chance of success, this practice leads to selection bias issues and other confounding factors when trying to evaluate outcomes of conservation projects [13]. In addition, baseline data are often missing in many situations where evaluations are conducted [66]. In the case of CAZ, while we did reconstruct REDD+ investments starting in 2007, the longer history of conservation and rural development investments that has occurred but for which information was missing, hindered our ability to control for these previous investments on conservation outcomes. Without clear control areas or before-after data, conservation organizations will be significantly challenged to measure their effects given the dynamic and diverse drivers of deforestation that can confound and mask the effects of conservation and development investments.

Even when a conservation program can document its own investments and outcomes, another challenge is how to control other investments made in the region by other actors. CAZ, like many other protected areas in Madagascar, represents a critical landscape for protection given the high levels of species endemism, valuable ecosystem services, and forest-dependent communities. The high value of Madagascar’s forests, combined with impending threats, have resulted in Madagascar receiving $240 million in conservation aid in the past decade from multiple investors and with dozens of development, health, and conservation organizations working in concurrent landscapes [1]. This study, for example, required two years of extensive research to review, document, and compile the best possible database of interventions for just one protected area in Madagascar, and yet we estimate the database is only 85% complete and the data limits the ability to do fine-scale evaluation. Partners working in the same landscape should be encouraged to systematically document and provide openly accessible information on their investments for the shared benefit of advancing conservation (and rural development) success. With the recent push by donors and governments to evaluate the effectiveness of investments and interventions [67], increasingly robust impact evaluations will be required, and projects/organizations will be tasked with similar challenges of information gathering to conduct these evaluations. Donors and governments can facilitate open and accessible information sharing by requiring practitioners to host these data on online data portals or publish with open web map services and by providing access to resources that facilitate collection and sharing of data.

## Conclusions

Our study assessed trends in fires and deforestation in CAZ from 2007 through 2014 and the impact of investments on forest conservation outcomes. Like many tropically biodiverse locations, CAZ represents a complex landscape with a long history of investments by multiple stakeholders. CAZ is also located in a nation with inconsistent governance due to decades of political instability. Our assessment of the impacts of investments on deforestation rates and fires suggested a significant effect on reducing fires with a more variable impact for reducing deforestation depending on the sample used and years of data. We also found that investments appeared to have reduced deforestation and forest fires during years of significant political instability. The relationship between dollars invested and conservation outcomes was stronger, particularly when there was sustained spending within the same fokontany (i.e. one to two years of previous funding to same fokontany). This suggests that conservation and rural development organizations are more likely to see positive conservation outcomes if they target the same area over multiple years.

While we compiled the best existing data for CAZ, our evaluation results could have been affected by a number of factors including missing data on other investments in the region, lack of a true baseline, and the volatile and dynamic nature of drivers of deforestation. While this study evaluated immediate conservation outcomes, future studies in this landscape should investigate the long-term sustainability of conservation and development investments many years post-investment [23]. Our recommendation for future evaluations of conservation interventions is to encourage strategic project design early in the evaluation process, first by isolating expected causal connections and then clearly documenting the location and timing of investments and outcomes. It will also be critical to move toward open data sharing between conservation and development organizations, governments, the private sector, and other investors working in the same landscape to more accurately measure shared conservation and development outcomes.

## Supporting information

S1 Table Summary statistics before and after log-transformation of dependent and treatment variables. We use a fixed effects linear panel model (Eq 1) to estimate the relationship between conservation investments and conservation outcomes. The underlying data had high skewness and were log-transformed. This greatly reduced the skewness of the data.
(DOCX)

S1 Fig Reducing deforestation skewness. Histogram of percent deforestation before (panel A) and after log-transformation (panel B).
(DOCX)

S2 Fig Relationships between investments and deforestation. A polynomial graph of the relationship between log-transformed dollars invested and log-transformed percent deforestation suggested a linear relationship. Thus, a linear relationship was estimated in Eq 1. Points represent underlying data and dashed line is fitted relationship.
(DOCX)

S3 Fig Reducing fire detections skewness. Histogram of fires before (panel A) and after log-transformation (panel B).
(DOCX)

S4 Fig Relationships between investments and fires. A polynomial graph of the relationship between log-transformed dollars invested and log-transformed number of fire detections suggested a linear relationship. Thus, a linear relationship was estimated in Eq 1. Points represent underlying data and dashed line is fitted relationship.
(DOCX)

S5 Fig Reducing investments skewness. Histogram of dollars invested before (panel A) and after log-transformation (panel B).
(DOCX)
