# Supplementary material for: Evaluating the effectiveness of conservation and development investments in reducing deforestation and fires in Ankeniheny-Zahemena Corridor, Madagascar
Source: PLoS One. 2017 Dec 21;12(12):e0190119. doi: 10.1371/journal.pone.0190119 (PMC5739477; doi:10.1371/journal.pone.0190119)
Supplement: S1 Table — We use a fixed effects linear panel model (Eq 1) to estimate the relationship between conservation investments and conservation outcomes. The underlying data had high skewness and were log-transformed. This greatly reduced the skewness of the data. (DOCX) [file pone.0190119.s001.docx]

|  | Percent deforestation | Number of fires | Dollars invested |
| --- | --- | --- | --- |
| *Before log-transformation* | | | |
| Mean | 0.96 | 2.53 | 2,607.62 |
| Standard deviation | 3.54 | 6.80 | 10.580.41 |
| Skewness | 10.96 | 4.94 | 8.67 |
| Kurtosis | 161.08 | 32.78 | 95.74 |
| *After log-transformation* | | | |
| Mean | 0.34 | 0.62 | 2.43 |
| Standard deviation | 0.62 | 0.92 | 3.82 |
| Skewness | 2.28 | 1.55 | 1.00 |
| Kurtosis | 8.78 | 4.75 | 2.14 |
