# Supplementary figures and images for: Evaluating the effectiveness of conservation and development investments in reducing deforestation and fires in Ankeniheny-Zahemena Corridor, Madagascar
Source: PLoS One. 2017 Dec 21;12(12):e0190119. doi: 10.1371/journal.pone.0190119 (PMC5739477; doi:10.1371/journal.pone.0190119)

**A B**


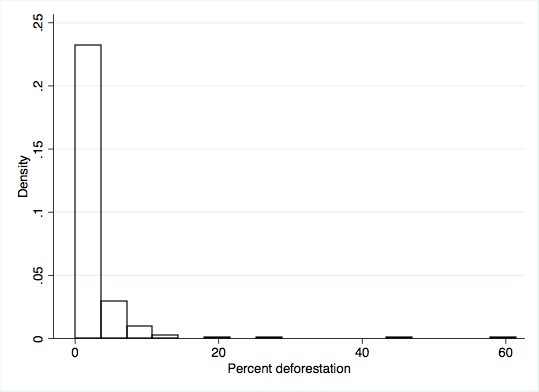

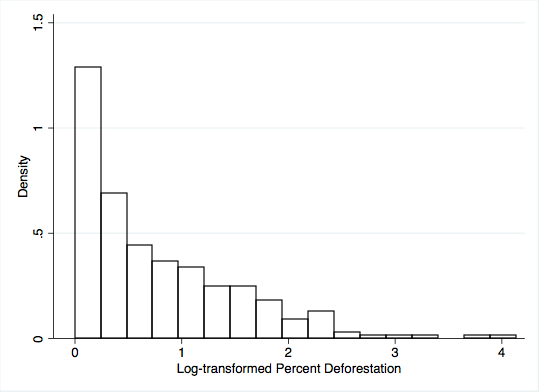

Supplement: S1 Fig — Histogram of percent deforestation before (panel A) and after log-transformation (panel B). (DOCX) [file pone.0190119.s002.docx]

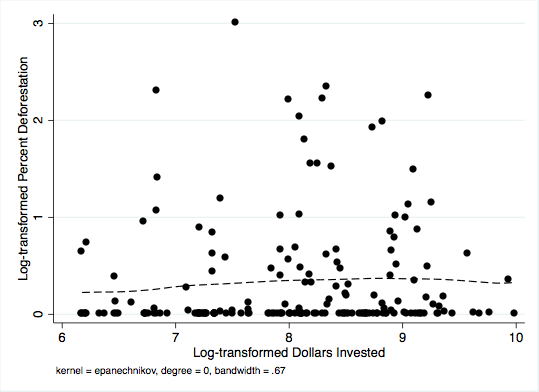

Supplement: S2 Fig — A polynomial graph of the relationship between log-transformed dollars invested and log-transformed percent deforestation suggested a linear relationship. Thus, a linear relationship was estimated in Eq 1. Points represent underlying data and dashed line is fitted relationship. (DOCX) [file pone.0190119.s003.docx]

**A B**


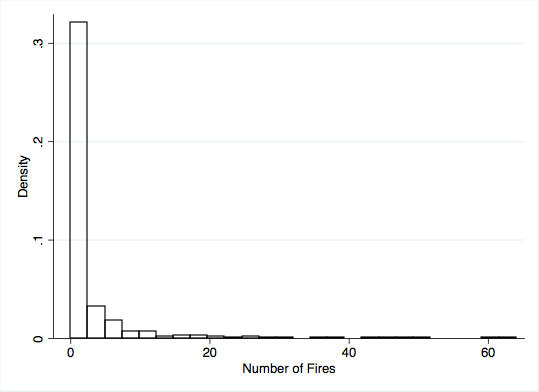

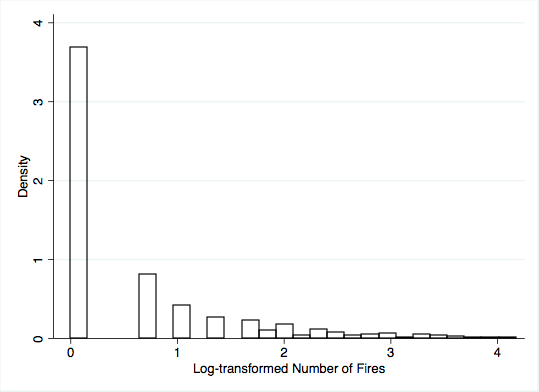

Supplement: S3 Fig — Histogram of fires before (panel A) and after log-transformation (panel B). (DOCX) [file pone.0190119.s004.docx]

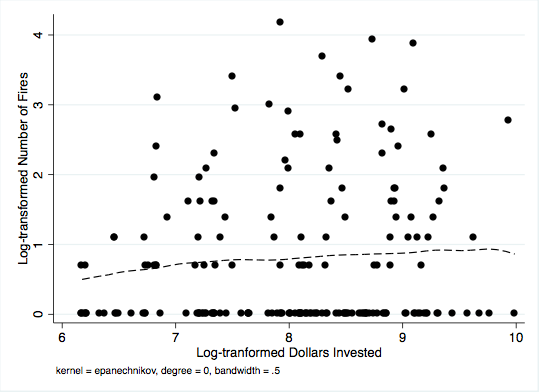

Supplement: S4 Fig — A polynomial graph of the relationship between log-transformed dollars invested and log-transformed number of fire detections suggested a linear relationship. Thus, a linear relationship was estimated in Eq 1. Points represent underlying data and dashed line is fitted relationship. (DOCX) [file pone.0190119.s005.docx]

**A B**


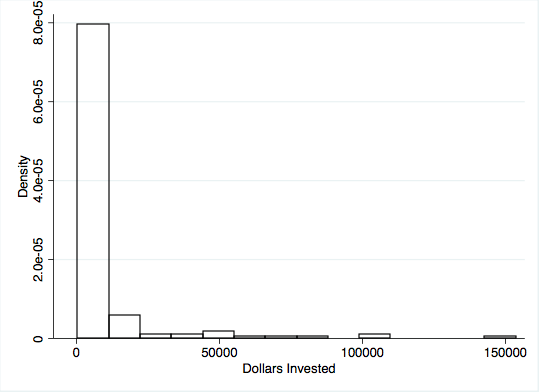

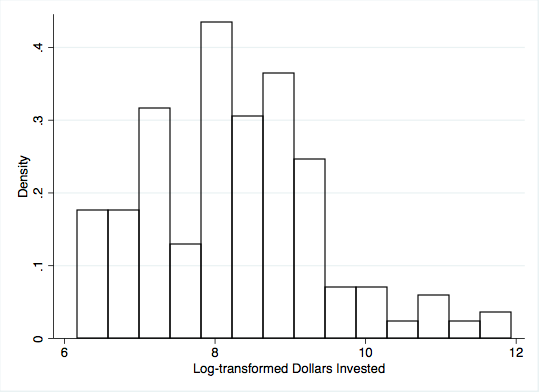

Supplement: S5 Fig — Histogram of dollars invested before (panel A) and after log-transformation (panel B). (DOCX) [file pone.0190119.s006.docx]
